# The computational model of nanofluid considering heat transfer and entropy generation across a curved and flat surface

**DOI:** 10.1038/s41598-023-46955-7

**Published:** 2023-11-16

**Authors:** Sayer Obaid Alharbi, Florentin Smarandache, Awatif M. A. Elsiddieg, Aisha M. Alqahtani, M. Riaz Khan, V. Puneeth, Nidhal Becheikh

**Affiliations:** 1 Mathematics Department, College of Science Al-Zulfi, Majmaah University, Majmaah, 11952 Saudi Arabia; 2 Mathematics, Physics, and Natural Science Division, The University of New Mexico, 705 Gurley Ave., Gallup, NM 87301 USA; 3 Department of Mathematics, College of Science and Humanities, Prince Sattam bin Abdul-Aziz University, Al-Kharj, 11942 Saudi Arabia; 4 Department of mathematical sciences, College of Science, Princess Nourah Bint Abdulrahman University, P. O. Box 84428, 11671 Riyadh, Saudi Arabia; 5 Department of Mathematics, Quaid-I-Azam University, Islamabad, 44000 Pakistan; 6 School of Sciences, CHRIST University, Delhi NCR, Ghaziabad, 201003 India; 7 College of Engineering, Northern Border University, 73222 Arar, Saudi Arabia

**Keywords:** Applied mathematics, Mathematics and computing

## Abstract

The entropy generation analysis for the nanofluid flowing over a stretching/shrinking curved region is performed in the existence of the cross-diffusion effect. The surface is also subjected to second-order velocity slip under the effect of mixed convection. The Joule heating that contributes significantly to the heat transfer properties of nanofluid is incorporated along with the heat source/sink. Furthermore, the flow is assumed to be governed by an exterior magnetic field that aids in gaining control over the flow speed. With these frameworks, the mathematical model that describes the flow with such characteristics and assumptions is framed using partial differential equations (PDEs). The bvp4c solver is used to numerically solve the system of non-linear ordinary differential equations (ODEs) that are created from these equations. The solutions of obtained through this technique are verified with the available articles and the comparison is tabulated. Meanwhile, the interpretation of the results of this study is delivered through graphs. The findings showed that the Bejan number was decreased by increasing Brinkman number values whereas it enhanced the entropy generation. Also, as the curvature parameter goes higher, the speed of the nanofluid flow diminishes. Furthermore, the increase in the Soret and Dufour effects have enhanced the thermal conduction and the mass transfer of the nanofluid.

## Introduction

The term nanofluid was coined in the year 1995 by Choi^1^ considering the fact of suspending the nanoparticles would enhance the capacity of the conduction of heat of the regular fluid. Nanofluid is a type of heat carrier that consists of a base fluid and metal particles of size ${10}^{-9}\; \mathrm{m}$. The nanoparticles minimal surface area allows the enrichment of heat/energy transfer. It is widely employed in various fields like automobiles, nuclear reactors, refrigerators, cooling of electronic appliances, and many other household essentials due to the nanofluid's increased thermal conductivity. Also, these nanofluids find application in various manufacturing industries. In this regard, Khan and Puneeth^2^ investigated how Brownian motion and thermophoresis affected the thermal characteristics of nanofluid. Sharma et al.^3^ gave a piece of collective information on the recent advances in machine learning that helps in utilizing it in the analysis of thermal properties of nanofluids. Zhang et al.^4^ analyzed the bioconvection process in enhancing the nanoparticle distribution in the nanofluid. Pramuanjaroenkij et al.^5^ completed a numerical study to understand the behavior of various thermal conductivity models for fluid. Further, Puneeth et al.^6^ came to the conclusion that as the Casson parameter is increased, jet speed of the Casson nanofluid decreases. A comparative analysis was carried out by Bheshti et al.^7^ for the flow of a nanofluid in an annulus. Alqahtani et al.^8^ observed an enhancement in the temperature profile of nanofluid flowing across a cylinder for higher radiation under the action of viscous dissipation. The flow of sodium alginate suspended with Al_2_O_3_ and Cu was studied by Nadeem et al.^9^ using the fuzzy hybrid nanofluid model. Atashafrooz^10,11^ studied the dynamics of water suspended with nanoparticles to understand the effectiveness of water with solid suspensions as the heat carrier. Kumar et al.^12^ performed irreversibility analysis of an unsteady non-Newtonian Micropolar fluid containing CNT to analyse its thermal features. Maiti et al.^13^ implemented the fractional order model to study the heat transfer properties of blood under the influence of thermochemical effects. Dhlamini et al.^14^ deliberated the phenomena of bioconvection in the flow of nanofluid past a hot surface. Atashafrooz et al.^15^ employed simulation to realize the pattern of the nanofluid flow in the interior of a trapezoidal enclosure. Furthermore, they^16^ considered the impression of Lorentz force on the velocity of the nanofluid flow using mathematical model and conclude that the increasing strength of Lorentz force decreases the velocity. More studies related to nanofluid can be read in^17–19^.

The level of irreversibility occurring during a process is determined by entropy generation which is described using the second law of thermodynamics. This law helps in minimizing the entropy generation that enables the identification of optimal engineering system designs. Meanwhile, entropy generation can be used as a criterion to examine the working of engineering appliances. Due to these advanced features of entropy generation, many researchers have theoretically analyzed its effect on the heat transport properties of nanofluid. For instance, Alsulami et al.^20^ studied the heat transfer in the swirling flow of nanofluid using modified Kriegger–Dougherty model. Sarada et al.^21^ analysed the impact of exponential heat source on the thermal properties of ternary nanofluid. Zhang et al.^22^ used the Joule heating to examine the impact of the magnetic field on the production of entropy in nanofluid. Khan et al.^23^ discussed the thermal features of a Casson nanofluid flowing across an expanding sheet. Alsulami et al.^24^ analysed the non-equilibrium conditions for the nanofluid flow comprised of $T{i}_{6}A{l}_{4}V$ and $AA7075$ nanoparticles. Punith et al.^25^ studied the impact fo the induced magnetic field that is generated due to the flow current. Huang et al.^26^ analysis the friction drag caused by the Lorentz effect on the flow of nanofluid across a curved surface. Shoaib et al.^27^ discussed the process of entropy generation in detail for the flow of nanofluid and hybrid nanofluid respectively across a stretching surface and rotating system.

The inclusion of nanoparticles into the fluid not only enhances its heat conduction capacity but also influences the fluid in many different aspects. For instance, the viscosity of the nanoparticles significantly increases based on its physical properties, density is often increased and as discussed earlier, the thermal conductance also increases. The difference in the thermal expansion coefficients between the nanoparticles and base fluid will cause thermal expansion induced convection which in turn affects the heat transfer. Jamshed et al.^28^ studied the entropy effect on the flow of second grade nanofluid. Atashafrooz et al.^29^ studied the entropy generation and the impact of Bejan number along with the analysis of the thermal features of nanofluid. Mandal et al.^30^ considered the features of heat transport of a nanofluid considering the Entropy generation with the existence of microorganisms. Oyelakin et al.^31^ obtained an optimized model for accurate estimation of entropy generation for the Casson nanofluid. Nayak et al.^32^ designed a 3D model to analyze the impact of radiation and the Entropy generation over the flow features and heat transfer of a nanofluid.

The addition of a temperature gradient at the borders causes a more dynamic and significant effect to be produced in the fluid flow. This non-homogeneity in the thermal distribution produces a buoyancy effect which alternatively will have an impact on the coupled fields of velocity and temperature in the medium. The knowledge of mixed, forced, and natural convective flow plays a very important role in the fluid dynamics point of view as well as in practical engineering applications. The mixed convection impact on the flow of hybrid nanofluid was analyzed by Xia et al.^33^ with multiple slips at the boundary. Dawar and Acharya^34^ studied the timed dependent flow of nanofluid influenced by a mixed convection. Wang and Xu^35^ obtained a very accurate solution for analyzing the influence of convection over the nanofluid flowing in a lid-driven cavity using the wavelet-homotopy method. Khan et al.^36^ demonstrated that as the mixed convection parameter increases, the velocity of the hybrid nanofluid decreases. Tian et al.^37^ showed that mixed convection impacts positively on the nanofluid flow in an inclined cavity. Wahid et al.^38^ concluded that the strength in the magnetic field diminished the velocity of the flow across a porous vertical cone. It was observed by Ketchate et al.^39^ that the nanoparticles of blade shape stabilized the convective flow. Mahmood et al.^40^ designed a mathematical model to interpret the heat flow and the motion of nanofluid in a square cavity. Further Muhammad et al.^41–43^ elaborated the use of openFOAM in analyzing the fluid properties.

The nanofluid flowing across a stretched sheet has a significant role in practical applications including the manufacturing of glass fiber, plastic film extraction, condensation of liquid films, paper production, etc. In these applications, a large amount of heating is involved and hence cooling of appliances becomes a mandatory process to maintain an optimum temperature. Thus, many scholars are actively working on analyzing the thermal/energy characteristics of the nanofluid motion across the stretching sheet and other various geometries. For instance, Reddy et al.^44^ studied the significance of radiation on the stagnation point flow of nanofluid over a curved surface. Abbas et al.^45^ completed the numerical investigation to estimate the heat transfer of time-dependent/unsteady flow of micropolar fluid flowing past a curved region. Qian et al.^46^ framed a mathematical model that described the significant role of the Lorentz force caused by the magnetic field on the micropolar fluid flowing across a curved stretching sheet. Naveen et al.^47^ incorporated the model of Cattaneo-Christov to design the heat flux in the flow of nanofluid going through a curved stretching sheet. Khan et al.^48^ considered the impact of gold nanoparticles on enhancing the thermal properties of blood flowing through a curved surface. Ashraf et al.^49^ examined the enhancement in the heat transfer rate of a fluid suspended with Al_2_O_3_ and Fe_3_O_4_ nanoparticles. Hayat et al.^50^ deliberate the entropy generation process in the nanofluid going through a curved region. To analyze the heat and mass transport of a nanofluid passing through a curved sheet, Imtiaz et al.^51^ created the energy equation using the Soret-Dufour model. Alblawi et al.^52^ applied the Buongiorno’s model to analyze the effect of the major slip mechanisms on the heat transport features of nanofluid.

The detailed literature review provided above indicated that the availability of resources on the flow of nanofluid past a curved stretching/shrinking surface is limited. Meanwhile, there were no resources available which can describe the influence of activation energy over the motion of nanofluid across a curved surface by considering the Soret and Dufour effect. Thus, the authors have incorporated these effects along with the Joule heating, second order velocity slip, heat source/sink radiation and the magnetic field effects. The practical applications of the Soret effects includes the isotope separation, purification of gases and liquids, isoelectric focusing, purification and analysis of proteins, biomolecules, and drug molecules, whereas the applications of Soret effect includes heat exchangers, combustion processes, cryogenic systems, and semiconductor processing. The mathematical model for the considered effects is constructed using PDEs, and this system of equations is subsequently translated to yield the proper system of ODEs. The solutions to the resulting system of ODEs are obtained using the MATLAB bvp4c tool, and the outcomes are displayed graphically using graphs.

## Basic governing equations

**Figure 1:**
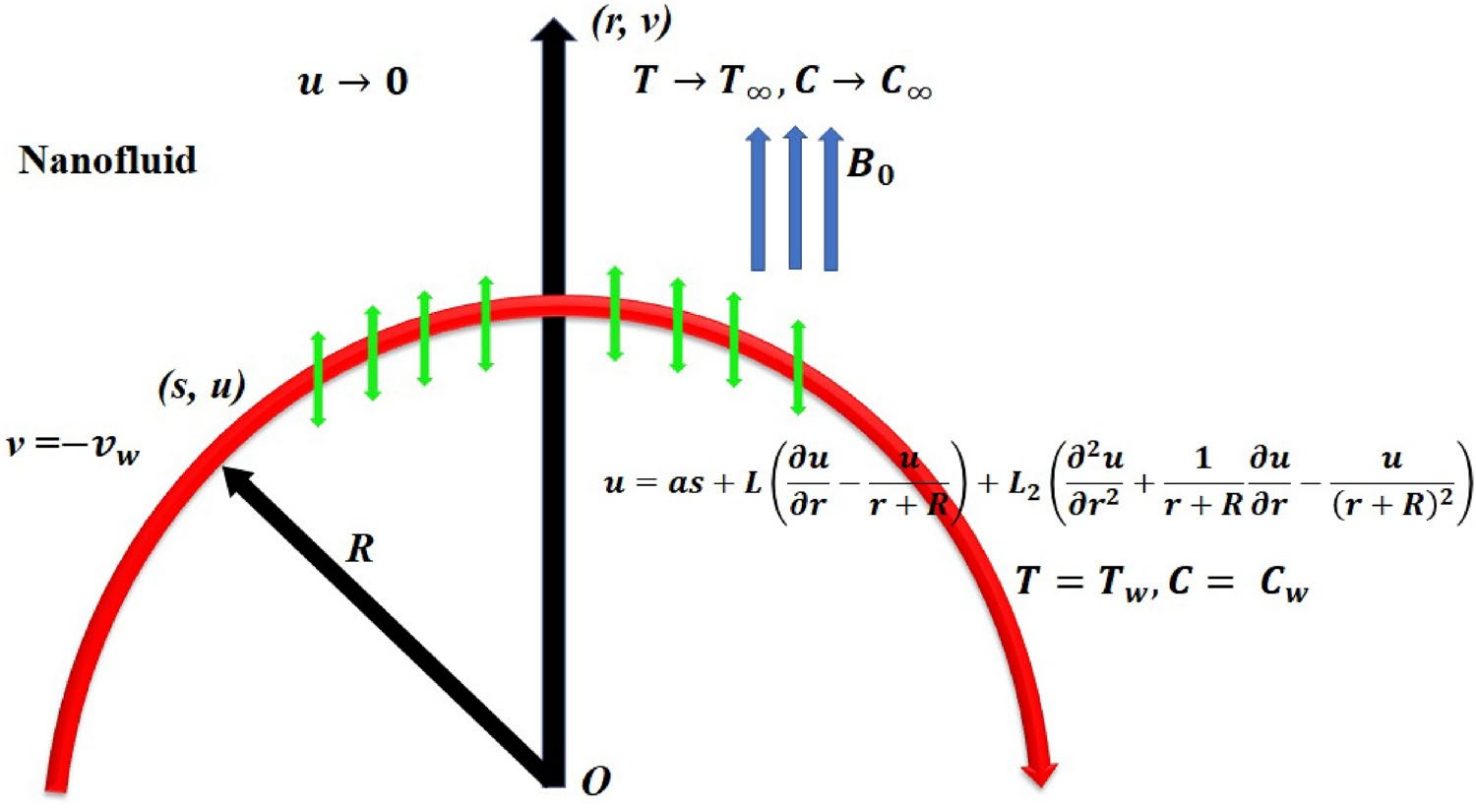
Schematic diagram of the problem displaying two-dimensional motion of the nanofluid across a curved stretching/shrinking surface.

Consider the dissipative mixed convective flow of a nanofluid across a stretching/shrinking curved sheet as shown in Fig. 1. The flow is two-dimensional and the fluid is incompressible including the effect of Joule heating, thermal radiation, second order velocity slip, activation energy and heat generation/absorption. The Dufour and Soret numbers were also appropriately considered in the energy and mass diffusion equations. The two directions, $r$ and $s$ were taken as being respectively vertical to the surface and along the surface with the stretching/shrinking and second order slip velocity $u=as+{L}_{1}\left(\frac{\partial u}{\partial r}-\frac{u}{r+R}\right)+{L}_{2}\left(\frac{{\partial }^{2}u}{\partial {r}^{2}}+\frac{1}{r+R}\frac{\partial u}{\partial r}-\frac{u}{{\left(r+R\right)}^{2}}\right),$ as well as the free stream velocity is $u\to 0$. Note that $a=0, a<0$ and $a>0$ correspondingly indicates the static, shrinking and stretching surface, whereas ${L}_{1}$ and ${L}_{2}$ respectively signifies the first and the second order slip coefficients. There was a fixed radial magnetic field with an intensity of ${B}_{0}$. Given these factors, the following are the governing boundary layer equations^53–55^.

1 $$\frac{\partial }{\partial r}\left[(r+R)v\right]+R\frac{\partial u}{\partial s}=0,$$

2 $$\frac{1}{\rho }\frac{\partial p}{\partial r}-\frac{{u}^{2}}{r+R}=0,$$

3 $$\begin{aligned} \frac{R}{r+R}\frac{\partial p}{\partial s}+\rho \left(v\frac{\partial u}{\partial r}+\frac{Ru}{r+R}\frac{\partial u}{\partial s}+\frac{uv}{r+R}\right) & =\mu \left(\frac{{\partial }^{2}u}{\partial {r}^{2}}+\frac{1}{r+R}\frac{\partial u}{\partial r}-\frac{u}{{\left(r+R\right)}^{2}}\right) \\ & \quad +g\rho {\beta }_{c}\left(T-{T}_{\infty }\right)+g\rho {\beta }_{T}\left(C-{C}_{\infty }\right)-\sigma {{B}_{0}}^{2}u, \end{aligned}$$

4 $$\begin{aligned} \left(v\frac{\partial T}{\partial r}+\frac{Ru}{r+R}\frac{\partial T}{\partial s}\right) & =\frac{k}{\rho {C}_{p}}\left(\frac{{\partial }^{2}T}{\partial {r}^{2}}+\frac{1}{r+R}\frac{\partial T}{\partial r}\right)+\frac{\sigma }{\rho {C}_{p}}{{B}_{0}}^{2}{u}^{2}+\frac{1}{\rho {C}_{p}}\left(\frac{{\partial }^{2}T}{\partial {r}^{2}}+\frac{1}{r+R}\frac{\partial T}{\partial r}\right)\frac{16{\sigma }^{*}{T}_{\infty }^{3}}{3{k}^{*}} \\ & \quad +\frac{\mu }{\rho {C}_{p}}{\left(\frac{\partial u}{\partial r}-\frac{u}{r+R}\right)}^{2}+\frac{{Q}_{0}}{\rho {C}_{p}}\left(T-{T}_{\infty }\right)+\frac{1}{\rho {C}_{p}}\frac{{D}_{m}{k}_{T}}{{c}_{s}}\left(\frac{{\partial }^{2}C}{\partial {r}^{2}}+\frac{1}{r+R}\frac{\partial C}{\partial r}\right), \end{aligned}$$

5 $$\left(v\frac{\partial C}{\partial r}+\frac{Ru}{r+R}\frac{\partial C}{\partial s}\right)={D}_{m}\left(\frac{{\partial }^{2}C}{\partial {r}^{2}}+\frac{1}{r+R}\frac{\partial C}{\partial r}\right)+\frac{{D}_{m}{c}_{s}{k}_{T}}{{T}_{m}}\left(\frac{{\partial }^{2}T}{\partial {r}^{2}}+\frac{1}{r+R}\frac{\partial T}{\partial r}\right)-\frac{{K}_{r}^{2}}{{C}_{\infty }} {\left(\frac{T}{{T}_{\infty }}\right)}^{n}{e}^{-\frac{{E}_{a}}{{K}_{1}T}}\left(C-{C}_{\infty }\right).$$

The related boundary conditions are specified as

6 $$\left.\begin{array}{l}\left.\begin{array}{c}u=as+{L}_{1}\left(\frac{\partial u}{\partial r}-\frac{u}{r+R}\right)+{L}_{2}\left(\frac{{\partial }^{2}u}{\partial {r}^{2}}+\frac{1}{r+R}\frac{\partial u}{\partial r}-\frac{u}{{\left(r+R\right)}^{2}}\right), v=-{v}_{w},\\ T={T}_{w}, C= {C}_{w}, at r=0\end{array}\right\}\\ u\to 0, \frac{\partial u}{\partial r}\to 0, T\to {T}_{\infty }, C\to {C}_{\infty } as r\to \infty \end{array}\right].$$

The following dimensionless transformations can be utilized to transform Eqs. (1–6) into a non-dimensional structure.

7 $$\left.\begin{array}{l}u=bs{f}^{\prime}\left(\eta \right), \eta =\sqrt{\frac{b}{v}} r, v=-\frac{R}{r+R}\sqrt{b\nu }f\left(\eta \right) , p=\rho {b}^{2}{s}^{2}P\left(\eta \right)\\ T=\theta \left(\eta \right)\left({T}_{w}-{T}_{\infty }\right)+{T}_{\infty }, C=\phi \left(\eta \right)\left({C}_{w}-{C}_{\infty }\right)+{C}_{\infty }\end{array}\right\}.$$

As a result, the following structure is assumed by the resulting non-dimensional equation.

8 $$\frac{\partial P}{\partial \eta }=\frac{1}{\eta +K}{f}^{\prime2},$$

9 $$\frac{2}{\eta +K}P={f}^{{\prime}{\prime}{\prime}}-\frac{1}{{\left(\eta +K\right)}^{2}}{f}^{\prime}+\frac{1}{\eta +K}{f}^{{\prime}{\prime}}-\frac{K}{\eta +K}{\left({f}^{\prime}\right)}^{2}+\frac{K}{\eta +K}f{f}^{{\prime}{\prime}}+\frac{K}{{\left(\eta +K\right)}^{2}}f{f}^{\prime}+\lambda \theta +{\lambda }^{*}\phi -{M}^{2}{f}^{\prime},$$

10 $$\frac{1}{Pr}\left(1+Rd\right)\left({\theta }^{{\prime}{\prime}}+\frac{1}{\eta +K}{\theta }^{\prime}\right)+\frac{K}{\eta +K}f{\theta }^{\prime}+{E}_{c}{\left({f}^{{\prime}{\prime}}-\frac{{f}^{\prime}}{\eta +K}\right)}^{2}+{{M}^{2}E}_{c}{\left({f}^{\prime}\right)}^{2}+Q\theta +Du\left(\phi ^{{\prime}{\prime}}+\frac{1}{\eta +K}\phi ^{\prime}\right)=0,$$

11 $${\phi }^{{{\prime}}{{\prime}}}+\frac{1}{\eta +K}{\phi }^{{{\prime}}}+Sc\left\{\frac{K}{\eta +K}f{\phi }^{{{\prime}}}+Sr\left({\theta }^{{{\prime}}{{\prime}}}+\frac{1}{\eta +K}{\theta }^{{{\prime}}}\right)-{\tau \left(1+\omega \theta \right)}^{n}Exp\left(-\frac{{E}_{1}}{1+\omega \theta }\right)\right\}=0,$$

We may remove the pressure $P$ from Eq. (9) based on Eq. (8). Therefore, the sum of Eqs. (8) and (9) can be expressed as.

12 $${f}^{iv}+\frac{2}{\eta +K}{f}^{{\prime}{\prime}{\prime}}-\frac{1}{{\left(\eta +K\right)}^{2}}{f}^{{\prime}{\prime}}+\frac{1}{{\left(\eta +K\right)}^{3}}{f}^{\prime}+\frac{K}{\eta +K}\left(f{f}^{{\prime}{\prime}{\prime}}-{f}^{\prime}{f}^{{\prime}{\prime}}\right)+\frac{K}{{\left(\eta +K\right)}^{2}}\left(f{f}^{{\prime}{\prime}}-{{f}^{\prime2}}\right)-\frac{K}{{\left(\eta +K\right)}^{3}}f{f}^{\prime}+\lambda \left(\frac{\theta }{\eta +K}+{\theta }^{\prime}\right)+{\lambda }^{*}\left(\frac{\phi }{\eta +K}+{\phi }^{\prime}\right){-M}^{2}\left({f}^{{\prime}{\prime}}+\frac{1}{\eta +K}{f}^{\prime}\right)=0,$$

By re-arranging Eqs. (10) and (11) we get

13 $$\left\{\frac{1}{Pr}\left(1+Rd\right)-Sr ScDu\right\}{\theta }^{{\prime}{\prime}}+\left\{\frac{1}{Pr}\left(1+Rd\right)\frac{1}{\eta +K}-Sr ScDu\frac{1}{\eta +K}+\frac{K}{\eta +K}f\right\}{\theta }^{\prime}+Q\theta +DuSc \frac{K}{\eta +K}f{\phi }^{\prime}+{E}_{c}{\left({f}^{{\prime}{\prime}}-\frac{{f}^{\prime}}{\eta +K}\right)}^{2}+{{M}^{2}E}_{c}{\left({f}^{\prime}\right)}^{2}+\tau Du{\left(1+\omega \theta \right)}^{n}Sc Exp\left(-\frac{{E}_{1}}{1+\omega \theta }\right)=0,$$

14 $${\phi }^{{{\prime}}{{\prime}}}+\frac{1}{\eta +K}{\phi }^{{{\prime}}}+Sc\left\{\frac{K}{\eta +K}f{\phi }^{{{\prime}}}+Sr\left({\theta }^{{{\prime}}{{\prime}}}+\frac{1}{\eta +K}{\theta }^{{{\prime}}}\right)-{\tau \left(1+\omega \theta \right)}^{n}Exp\left(-\frac{{E}_{1}}{1+\omega \theta }\right)\right\}=0,$$

The boundary conditions defined in (6) are non-dimensionalised to the following form:

15 $$\left.\begin{array}{l}f\left(0\right)=S, {f}^{\prime}\left(0\right)=\gamma +{ \varepsilon }_{1}\left\{{f}^{{\prime}{\prime}}\left(0\right)+\frac{{f}^{\prime}\left(0\right)}{K}\right\}+{ \varepsilon }_{2}\left\{{f}^{{\prime}{\prime}{\prime}}\left(0\right)+\frac{{f}^{{\prime}{\prime}}\left(0\right)}{K}-\frac{{f}^{\prime}\left(0\right)}{{K}^{2}}\right\}, \\ \theta \left(\eta \right)=1, \phi \left(\eta \right)=1\\ {f}^{\prime}\left(\eta \right)=0, {f}^{{\prime}{\prime}}\left(\eta \right)=0, \theta \left(\eta \right)=0, \phi \left(\eta \right)=0 as \eta \to \infty .\end{array}\right\}.$$

Below are definitions for the dimensionless parameters that result from Eqs. (12–15).

16 $$\left.\begin{array}{l}K=R\sqrt{\frac{a}{\nu }}, {M}^{2}=\frac{\sigma {{B}_{0}}^{2}}{a\rho },\lambda =\frac{{G}_{r}}{{Re}_{x}^{2}} , {\lambda }^{*}=\frac{{G}_{r}^{*}}{{Re}_{x}^{2}} Pr=\frac{\rho \nu {C}_{p}}{k},{E}_{c}=\frac{{u}^{2}}{{C}_{p}({T}_{w}-{T}_{\infty })},\\ Rd=\frac{16{\sigma }^{*}{T}_{\infty }^{3}}{3k{k}^{*}}, Du=\frac{{D}_{m}{k}_{T}\left({C}_{w}-{C}_{\infty }\right)}{{c}_{s}\left(\mu {C}_{p}\right)\left({T}_{w}-{T}_{\infty }\right)}, Sc=\frac{{\nu }_{f}}{{D}_{m}},Sr=\frac{{D}_{m}{{c}_{s}k}_{T}\left({T}_{w}-{T}_{\infty }\right)}{{T}_{m}\nu \left({C}_{w}-{C}_{\infty }\right)} ,\gamma =\frac{a}{b}\\ \tau =\frac{{K}_{r}^{2}}{{C}_{\infty }b}, \omega =\frac{{T}_{w}-{T}_{\infty }}{{T}_{\infty }}, {E}_{1}=\frac{{E}_{a}}{{k}_{1}{T}_{\infty }},{ \varepsilon }_{1}={ L}_{1}\sqrt{\frac{a}{\nu }},{ \varepsilon }_{2}={ L}_{2}\frac{a}{\nu }, Q=\frac{{Q}_{0}}{a\rho {C}_{p}}.\end{array}\right\}.$$

## Entropy generation modeling

The entropy generation is defined as

17 $${S}_{gen}=\frac{k}{{T}_{\infty }^{2}}\left\{1+\frac{16{\sigma }^{*}{T}_{\infty }^{3}}{3k{k}^{*}}\right\}{\left(\frac{\partial T}{\partial r}\right)}^{2}+\frac{\mu }{{T}_{\infty }}{\left(\frac{\partial u}{\partial r}+\frac{u}{R+r}\right)}^{2}+\frac{\sigma {B}_{0}^{2}}{{T}_{\infty }}{u}^{2}+\frac{R{D}_{m}}{{C}_{\infty }}{\left(\frac{\partial C}{\partial r}\right)}^{2}+\frac{R{D}_{m}}{{T}_{\infty }}\left(\frac{\partial T}{\partial r}\frac{\partial C}{\partial r}\right).$$

In above equation, $\frac{k}{{T}_{\infty }^{2}}\left\{1+\frac{16{\sigma }^{*}{T}_{\infty }^{3}}{3k{k}^{*}}\right\}{\left(\frac{\partial T}{\partial r}\right)}^{2}$ represents the heat transfer irreversibility,

$\frac{\mu }{{T}_{\infty }}{\left(\frac{\partial u}{\partial r}+\frac{u}{R+r}\right)}^{2}$ represents the viscous dissipation irreversibility,

$\frac{\sigma {B}_{0}^{2}}{{T}_{\infty }}{u}^{2}$ represents the Joule heating irreversibility,

$\frac{R{D}_{m}}{{C}_{\infty }}{\left(\frac{\partial C}{\partial r}\right)}^{2}+\frac{R{D}_{m}}{{T}_{\infty }}\left(\frac{\partial T}{\partial r}\frac{\partial C}{\partial r}\right)$ represents the mass transfer irreversibility.

Keep in mind that $R$ is the universal gas constant.

Applying Eq. (7) results in the dimensionless version of Eq. (17), which may be expressed as

18 $${N}_{G}=\left(1+\frac{4}{3}Rd\right)\omega {{\theta }^{\prime}}^{2}+{B}_{r}{\left({f}^{{\prime}{\prime}}+\frac{1}{\eta +K}{f}^{\prime}\right)}^{2}+M{B}_{r}{{f}^{\prime}}^{2}+H\frac{{\omega }_{1}}{\omega }{{\phi }^{\prime}}^{2}+H{\theta }^{\prime}{\phi }^{\prime},$$

where the definitions of ${N}_{G}, {B}_{r}, H , \omega$ and ${\omega }_{1}$ are given below.

19 $${N}_{G}=\frac{{T}_{\infty }\nu {S}_{G}}{bk\Delta T}, {B}_{r}=\frac{\mu {b}^{2}{s}^{2}}{k\Delta T}, H=\frac{R{D}_{m}\left({C}_{w}-{C}_{\infty }\right)}{k}, \omega =\frac{{T}_{w}-{T}_{\infty }}{{T}_{\infty }}=\frac{\Delta T}{{T}_{\infty }}, {\omega }_{1}=\frac{{C}_{w}-{C}_{\infty }}{{C}_{\infty }}=\frac{\mathrm{\Delta C}}{{C}_{\infty }},$$

The definition of the dimensionless Bejan number formula is

20 $$Be=\frac{\mathrm{Entropy \; generation \; associated \; to \; heat \; and \; mass\; transfer}}{\mathrm{Total \; entropy \; generation}},$$

This implies that.

21 $$Be=\frac{\left(1+\frac{4}{3}Rd\right)\omega {{\theta }^{\prime}}^{2}+H\frac{{\omega }_{1}}{\omega }{{\phi }^{\prime2}}+H{\theta }^{\prime}{\phi }^{\prime}}{\left(1+\frac{4}{3}Rd\right)\omega {{\theta }^{\prime2}}+{B}_{r}{\left({f}^{{\prime}{\prime}}+\frac{1}{\eta +K}{f}^{\prime}\right)}^{2}+M{B}_{r}{{f}^{\prime 2}}+H\frac{{\omega }_{1}}{\omega }{{\phi }^{\prime}}^{2}+H{\theta }^{\prime}{\phi }^{\prime}},$$

## Solution method

The above dimensionless Eqs. (12–15) form boundary value problem and hence these equations are changed to a system of equations such that it forms an initial value problem. This enables us to implement the bvp4c numerical method using MATLAB where the interval of integration is assumed to be in the range 0–5 about the mesh point $70$. Further, the accuracy is set to ${10}^{-6}$ and the following relations are described to perform the conversion to initial value problem:

$$f\left(\eta \right)=y\left(1\right), {f}^{\prime}\left(\eta \right)=y\left(2\right), {f}^{{\prime}{\prime}}\left(\eta \right)= y\left(3\right), {f}^{{\prime}{\prime}{\prime}}\left(\eta \right)=y\left(4\right), {f}^{{\prime}v}\left(\eta \right)={yy}_{1},$$

$$\theta \left(\eta \right)=y\left(5\right), {\theta }^{\prime}\left(\eta \right)=y\left(6\right), {\theta }^{{\prime}{\prime}}\left(\eta \right)={yy}_{2}$$

$$\phi \left(\eta \right)=y\left(7\right), {\phi }^{\prime}\left(\eta \right)=y\left(8\right), {\phi }^{{\prime}{\prime}}\left(\eta \right)={yy}_{3}$$

we could rewrite the resulting Eqs. (12)–(15) as

22 $${yy}_{1}=-\frac{2}{\eta +K}y\left(4\right)+\frac{1}{{\left(\eta +K\right)}^{2}}y\left(3\right)-\frac{1}{{\left(\eta +K\right)}^{3}}y\left(2\right)-\frac{K}{\eta +K}\left(y\left(1\right)y\left(4\right)-y\left(2\right)y\left(3\right)\right)-\frac{K}{{\left(\eta +K\right)}^{2}}\left(y\left(1\right)y\left(3\right)-{\left(y\left(2\right)\right)}^{2}\right)+\frac{K}{{\left(\eta +K\right)}^{3}}y\left(1\right)y\left(2\right)-\lambda \left(\frac{y\left(5\right)}{\eta +K}+y\left(6\right)\right)+{\lambda }^{*}\left(\frac{y\left(7\right)}{\eta +K}+y\left(8\right)\right)-{M}^{2}\left(y\left(3\right)+\frac{1}{\eta +K}y\left(2\right)\right),$$

23 $${yy}_{2}=-\frac{Pr}{\left\{ \left(1+Rd\right)-Sc Sr Du\right\}}\left[\left\{\frac{1}{Pr}\left(1+Rd\right)\frac{1}{\eta +K}-Sr ScDu\frac{1}{\eta +K}+\frac{K}{\eta +K}y\left(1\right)\right\}y\left(6\right)-Qy\left(5\right)-Du Sc\frac{K}{\eta +K}y\left(1\right)y\left(8\right)-{E}_{c}{\left(y\left(3\right)-\frac{1}{\eta +K}y\left(2\right)\right)}^{2}-{{M}^{2}E}_{c}{\left(y\left(2\right)\right)}^{2}-\tau Du{\left(1+\omega y\left(5\right)\right)}^{n}Sc Exp\left(-\frac{{E}_{1}}{1+\omega y\left(5\right)}\right)\right],$$

24 $${yy}_{3}=-\frac{1}{\eta +K}y\left(8\right)-Sc\left\{\frac{K}{\eta +K}y\left(1\right)y\left(8\right)+Sr\left({yy}_{2}+\frac{1}{\eta +K}y\left(6\right)\right)-{\tau \left(1+\omega y\left(5\right)\right)}^{n}Exp\left(-\frac{{E}_{1}}{1+\omega y\left(5\right)}\right)\right\}.$$

The boundary conditions corresponding to (15) will take the following form:

25 $$\left.\begin{array}{l}y\left(1\right)-S=0, y\left(2\right)-\gamma -{ \varepsilon }_{1}\left\{y\left(3\right)+\frac{y\left(2\right)}{K}\right\}-{ \varepsilon }_{2}\left\{y\left(4\right)+\frac{y\left(3\right)}{K}-\frac{y\left(2\right)}{{K}^{2}}\right\}, \\ y\left(5\right)=1, y\left(7\right)=1 at \eta =0 \\ y\left(2\right)=0, y\left(3\right)=0, y\left(5\right)=0, y\left(7\right)=0 as \eta \to \infty \end{array}\right\},$$

## Results and discussion

**Figure 2:**
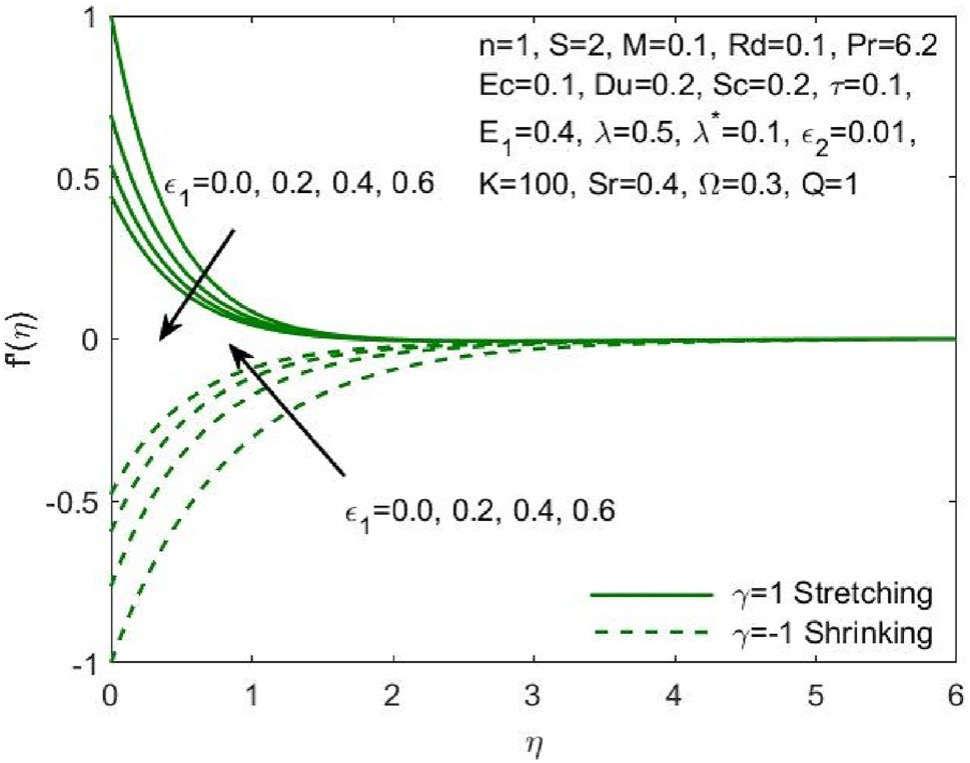
Influence of ${\varepsilon }_{1}$ on the ${f}^{\prime}\left(\eta \right)$.

**Figure 3:**
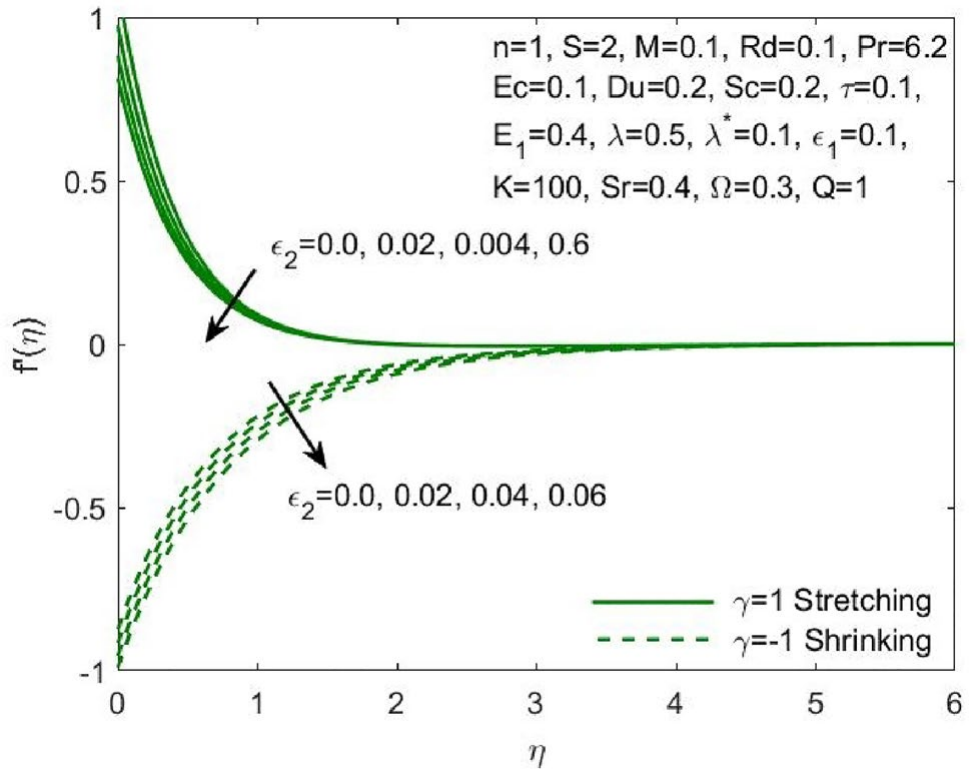
Influence of ${\varepsilon }_{2}$ on the ${f}^{\prime}\left(\eta \right)$.

**Figure 4:**
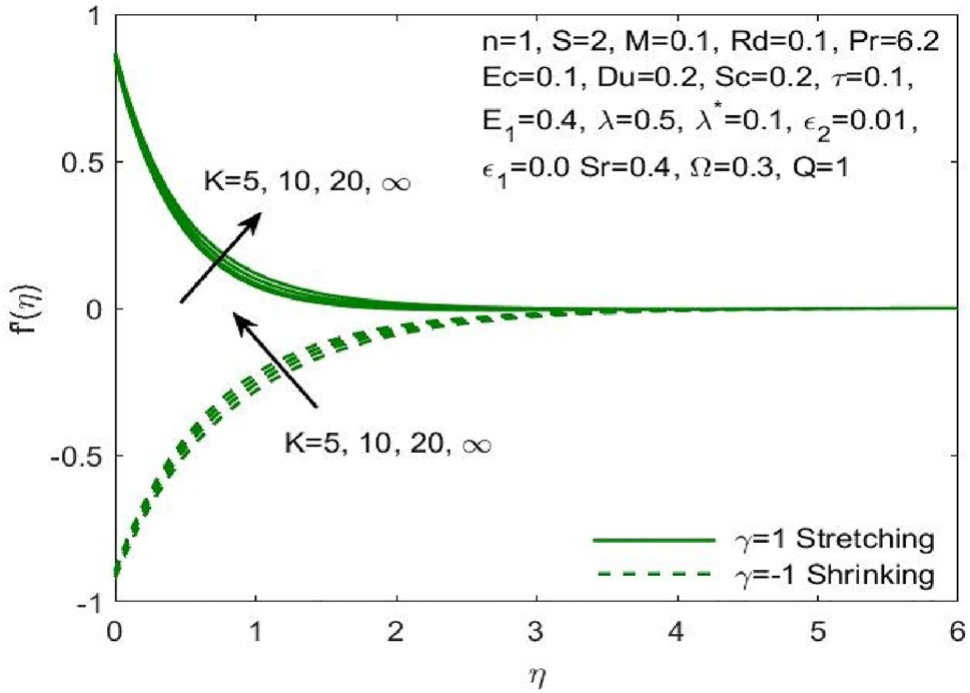
Influence of $K$ on the ${f}^{\prime}\left(\eta \right)$.

**Figure 5:**
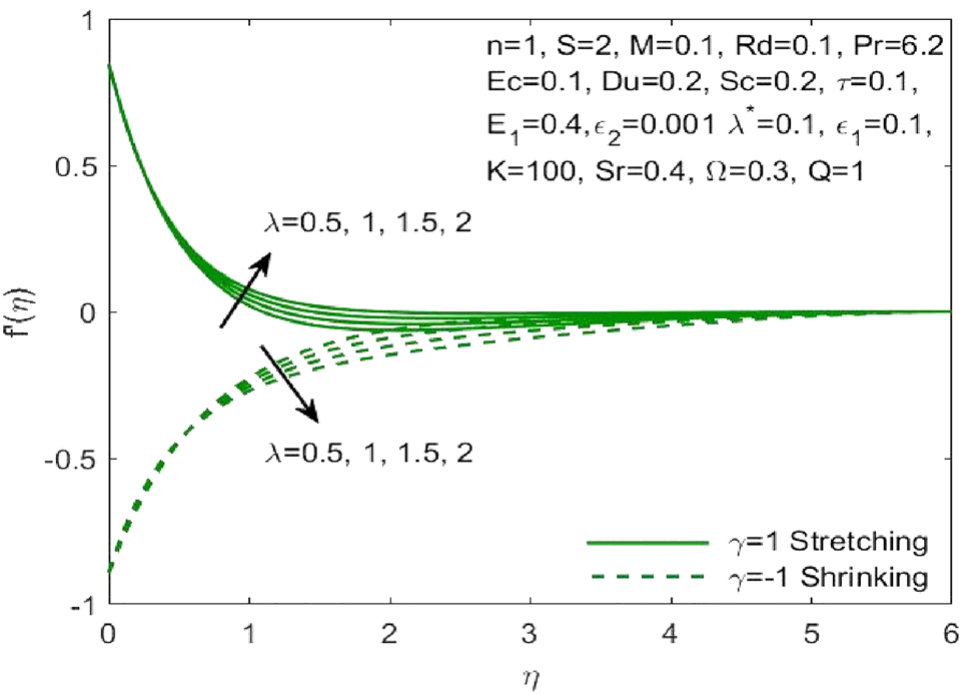
Influence of $\lambda$ on the ${f}^{\prime}\left(\eta \right)$.

**Figure 6:**
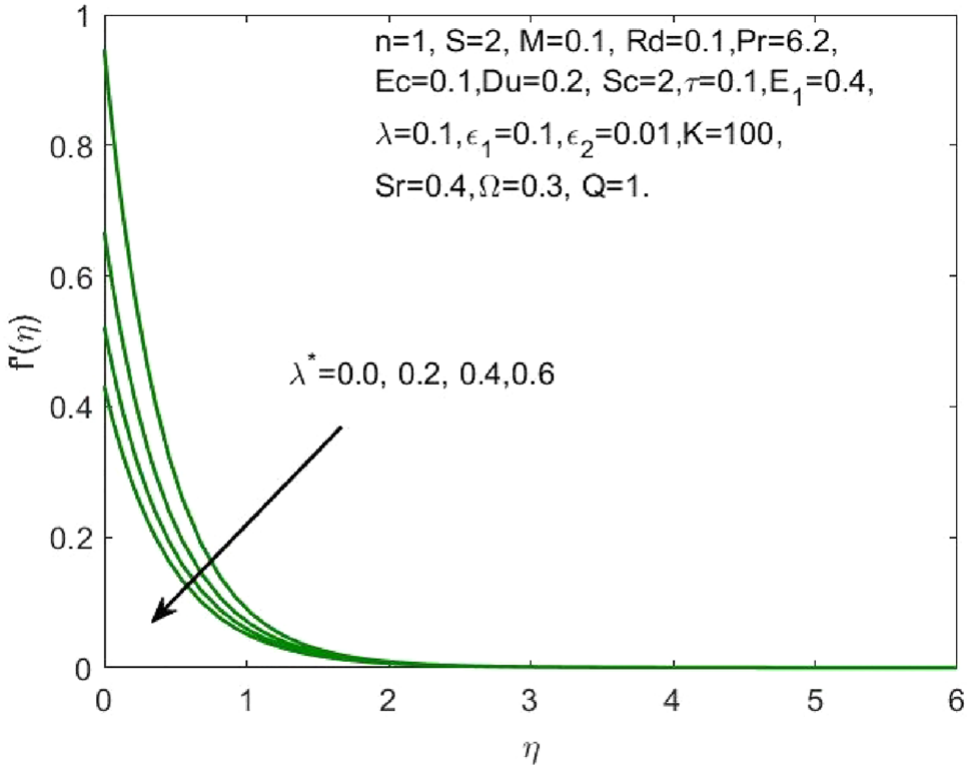
Influence of ${\lambda }^{*}$ on the ${f}^{\prime}\left(\eta \right)$.

**Figure 7:**
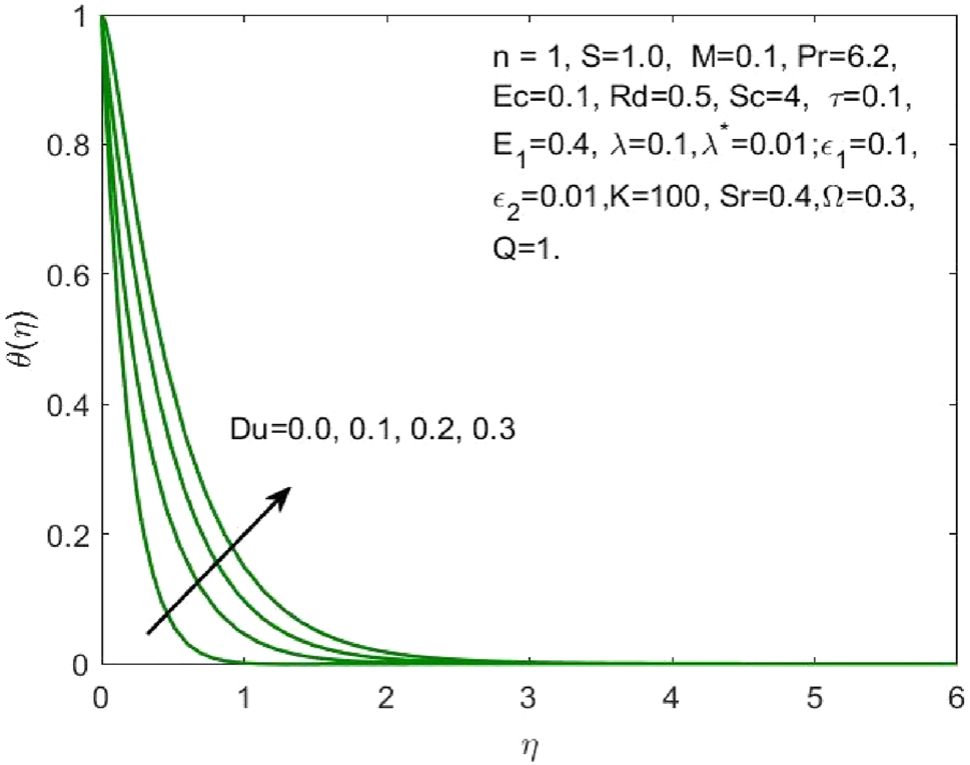
Influence of $Du$ on the $\theta \left(\eta \right)$.

**Figure 8:**
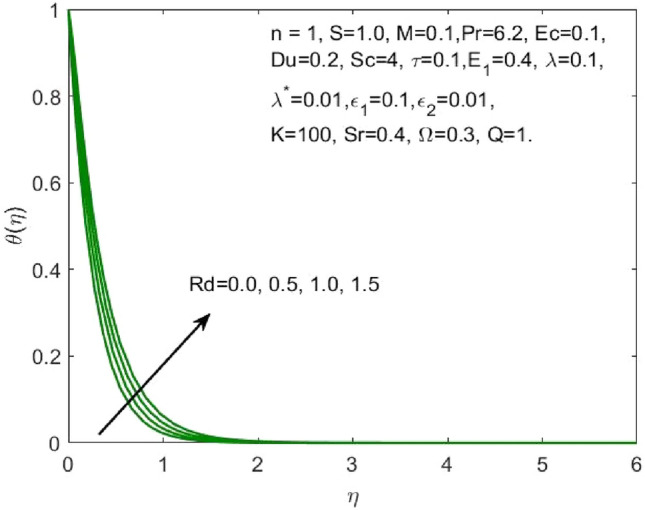
Influence of $Rd$ on the $\theta \left(\eta \right)$.

**Figure 9:**
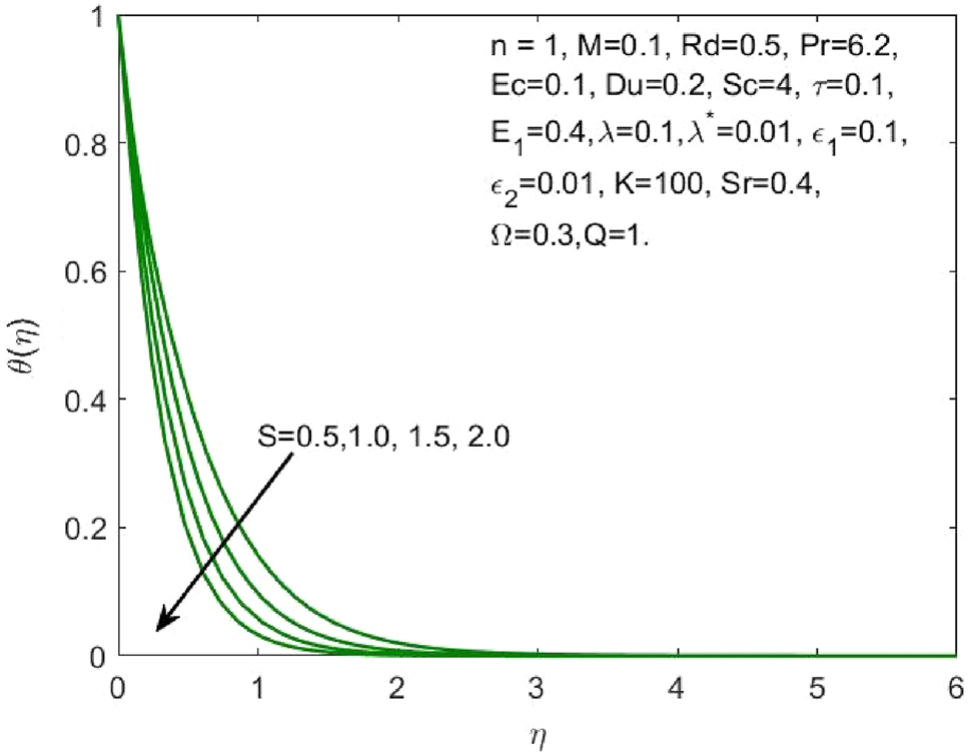
Influence of $S$ on the $\theta \left(\eta \right)$.

**Figure 10:**
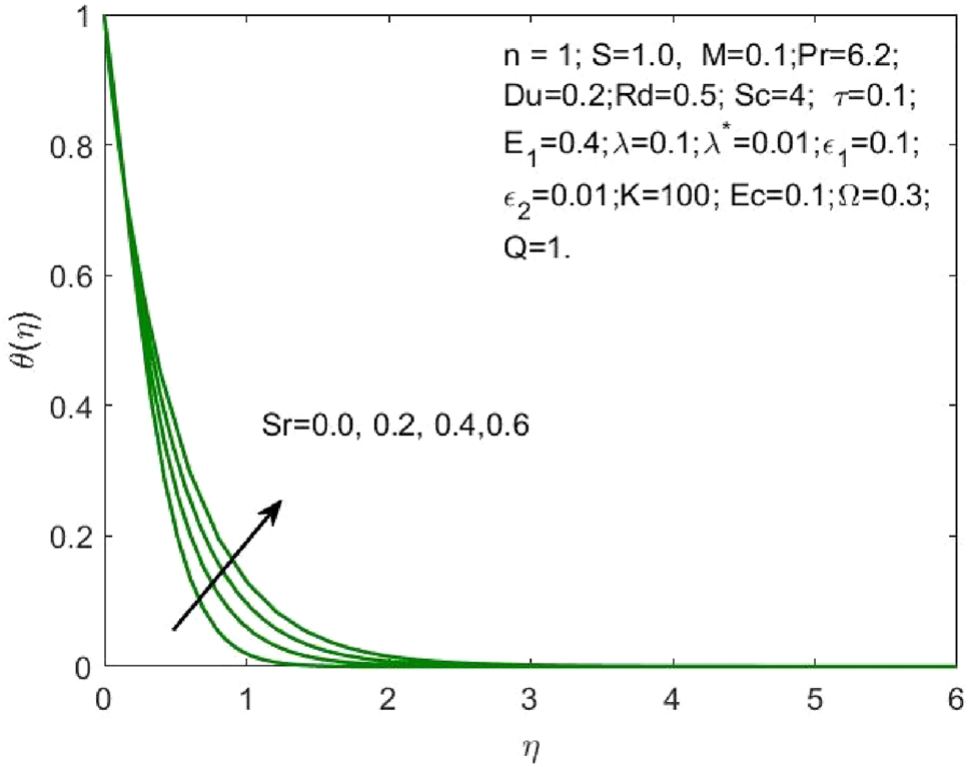
Influence of $Sr$ on the $\theta \left(\eta \right)$.

**Figure 11:**
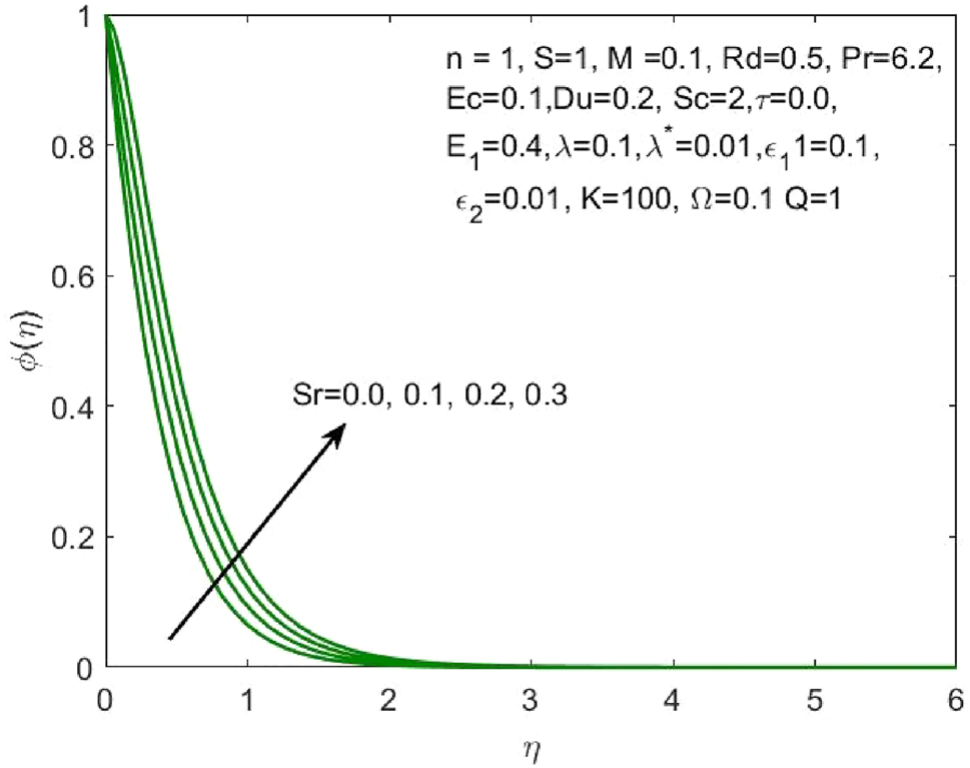
Influence of $Sr$ on the $\phi \left(\eta \right)$.

**Figure 12:**
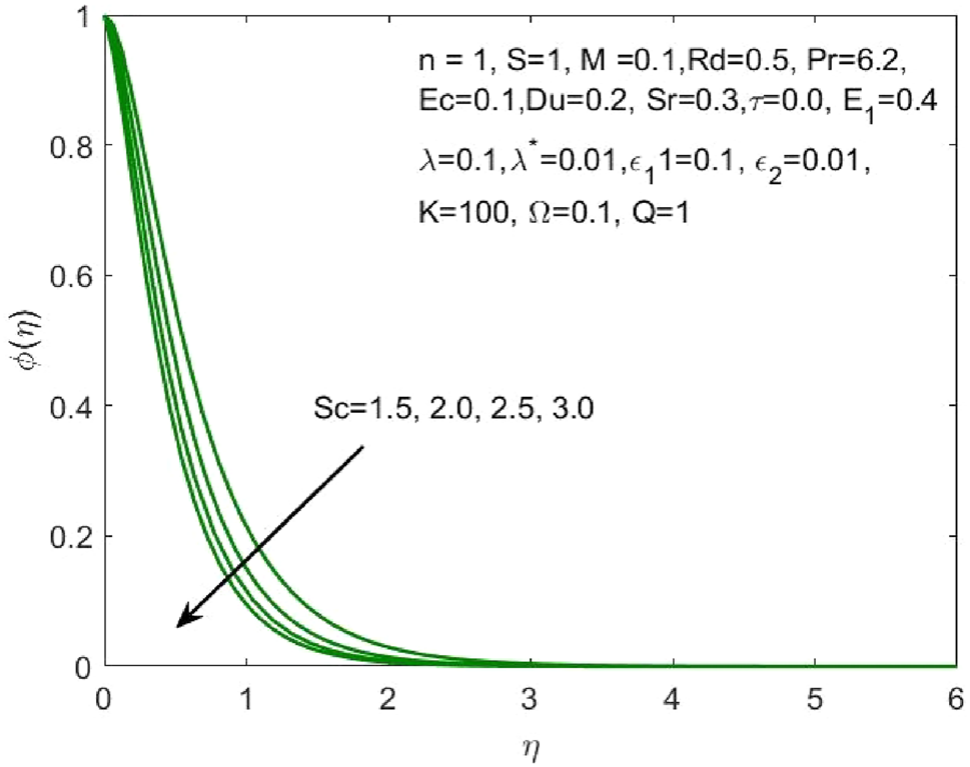
Influence of $Sc$ on the $\phi \left(\eta \right)$.

**Figure 13:**
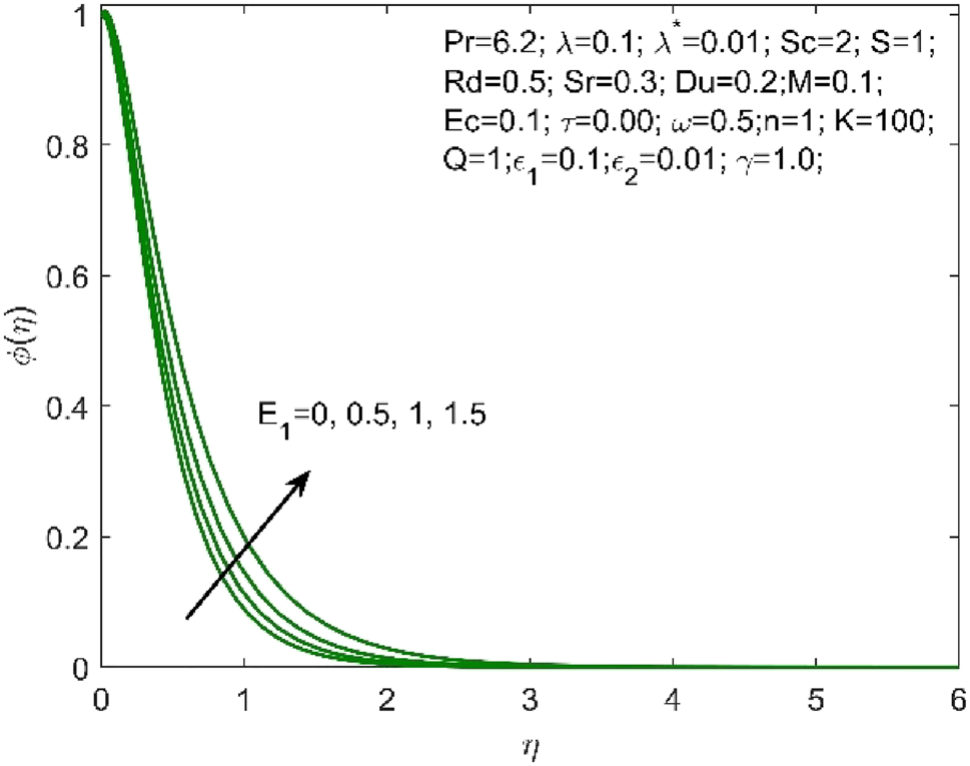
Influence of ${E}_{1}$ on the $\phi \left(\eta \right)$.

**Figure 14:**
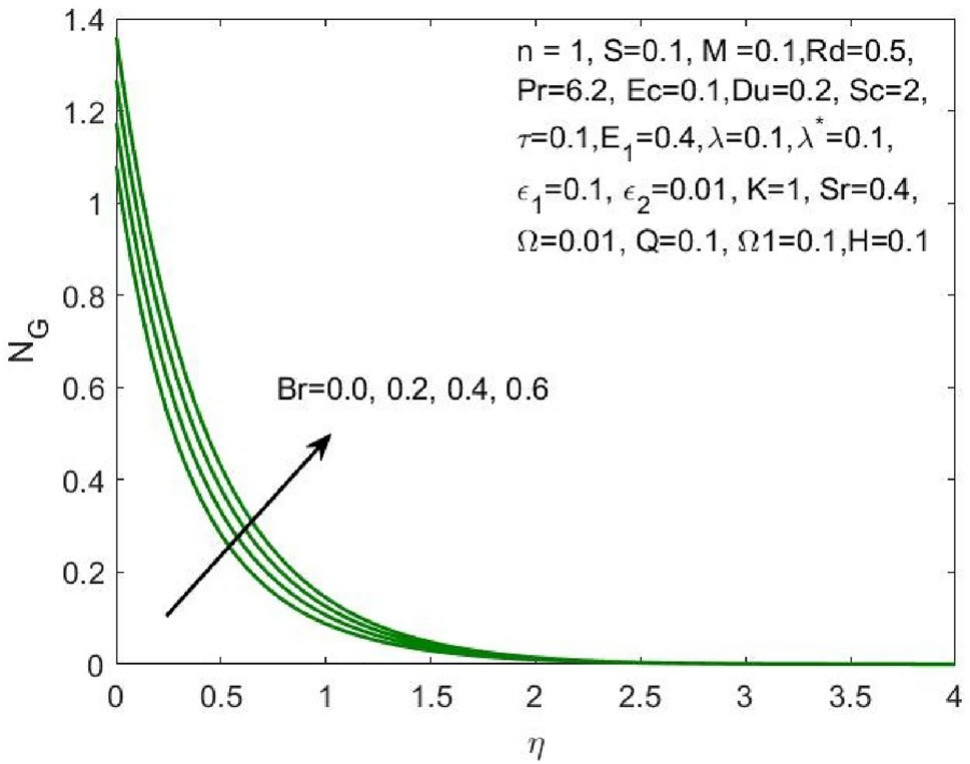
Influence of $Br$ on the ${N}_{G}$.

**Figure 15:**
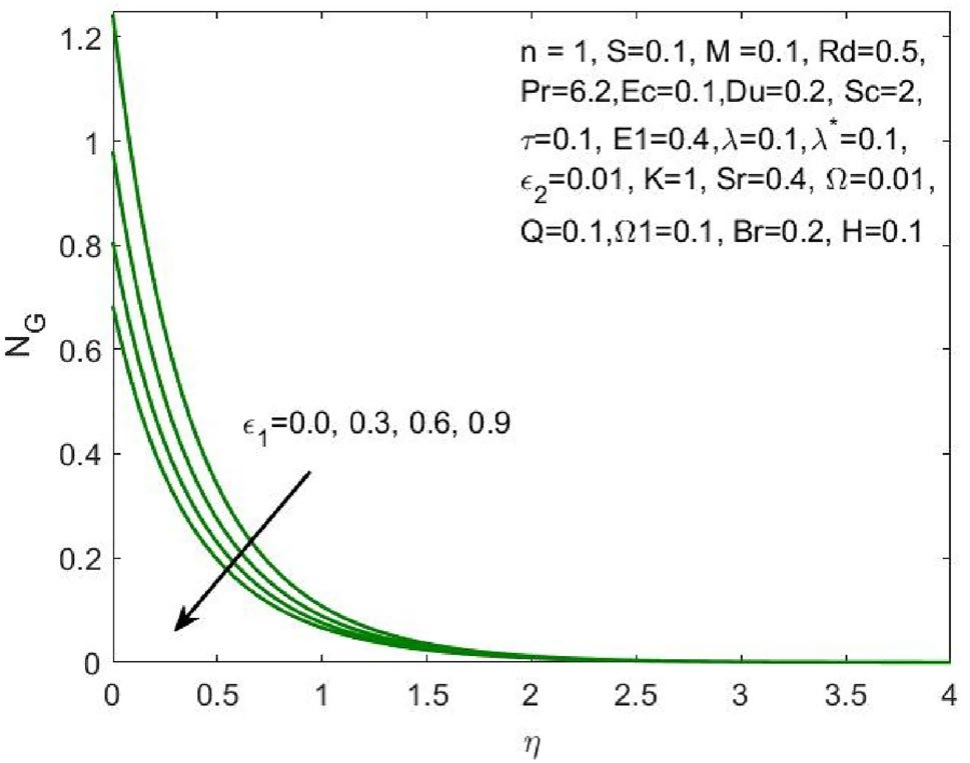
Influence of ${\varepsilon }_{1}$ on the ${N}_{G}$.

**Figure 16:**
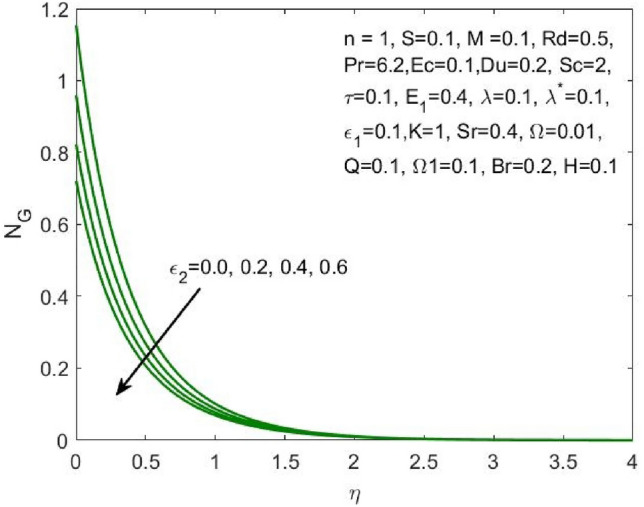
Influence of ${\varepsilon }_{2}$ on the ${N}_{G}$.

**Figure 17:**
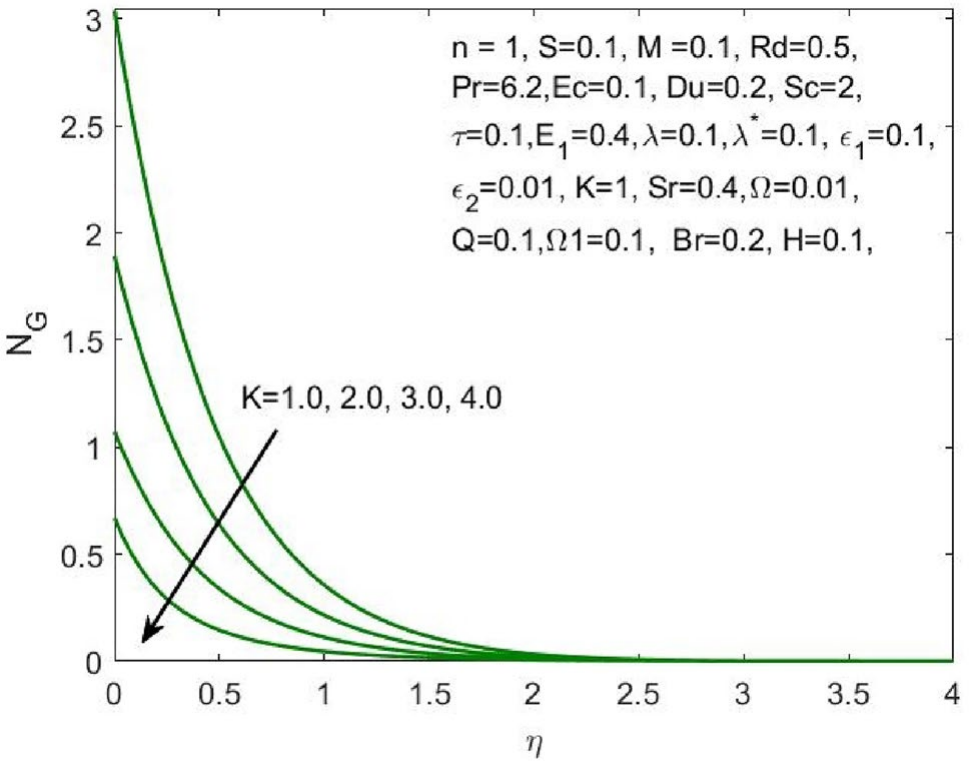
Influence of $K$ on the ${N}_{G}$.

**Figure 18:**
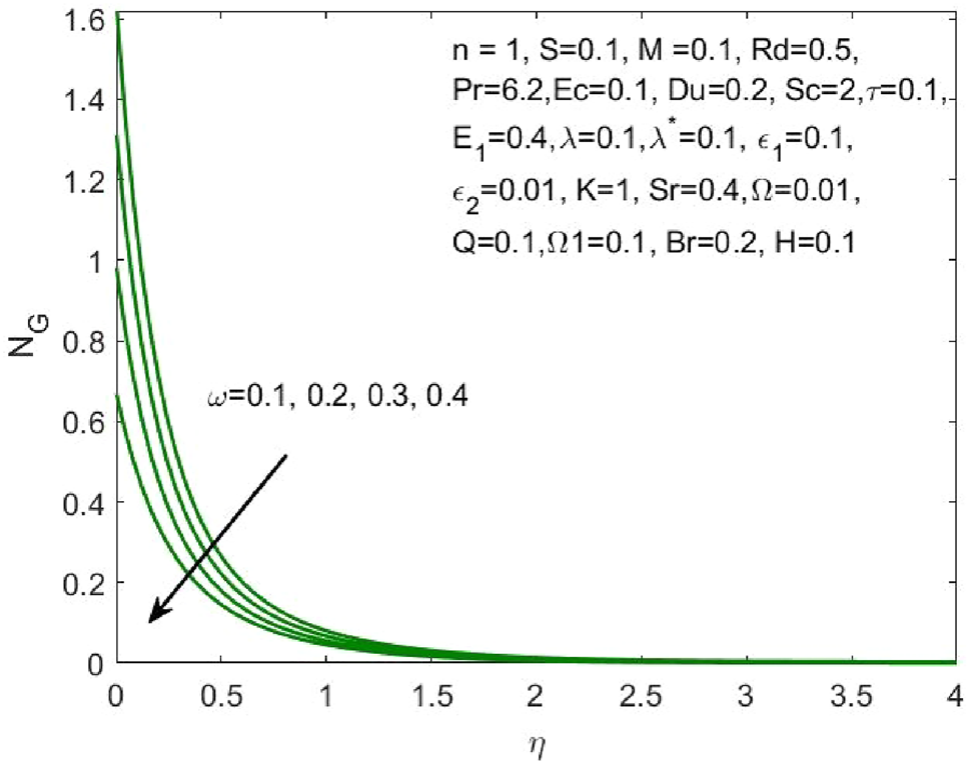
Influence of $\omega$ on the ${N}_{G}$.

**Figure 19:**
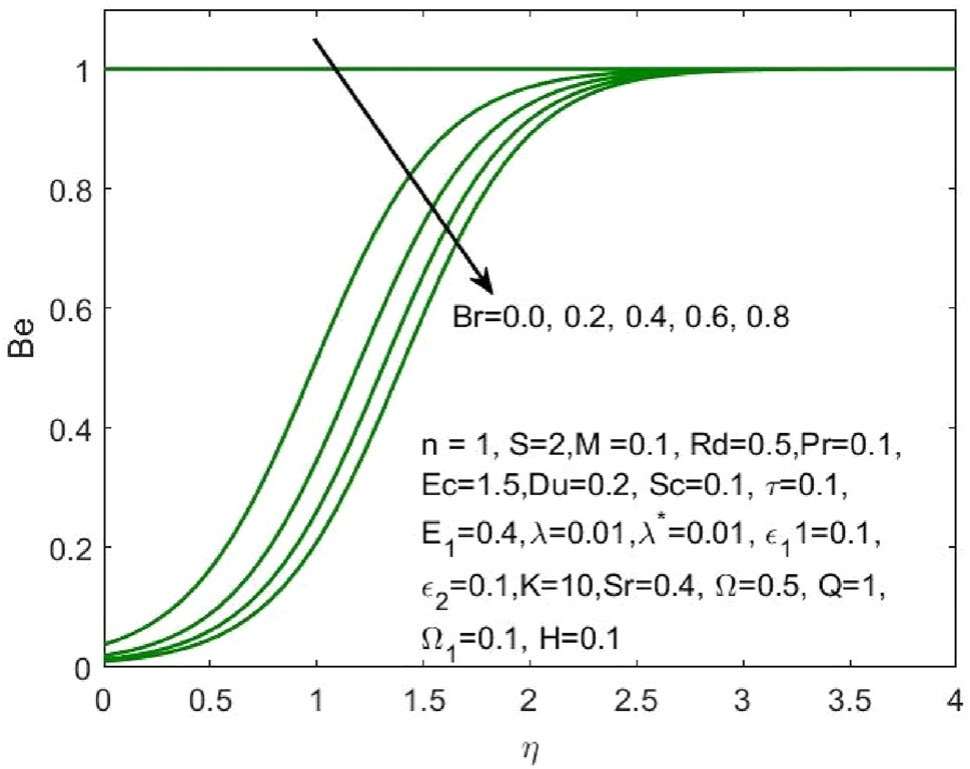
Influence of $Br$ on the $Be$.

**Figure 20:**
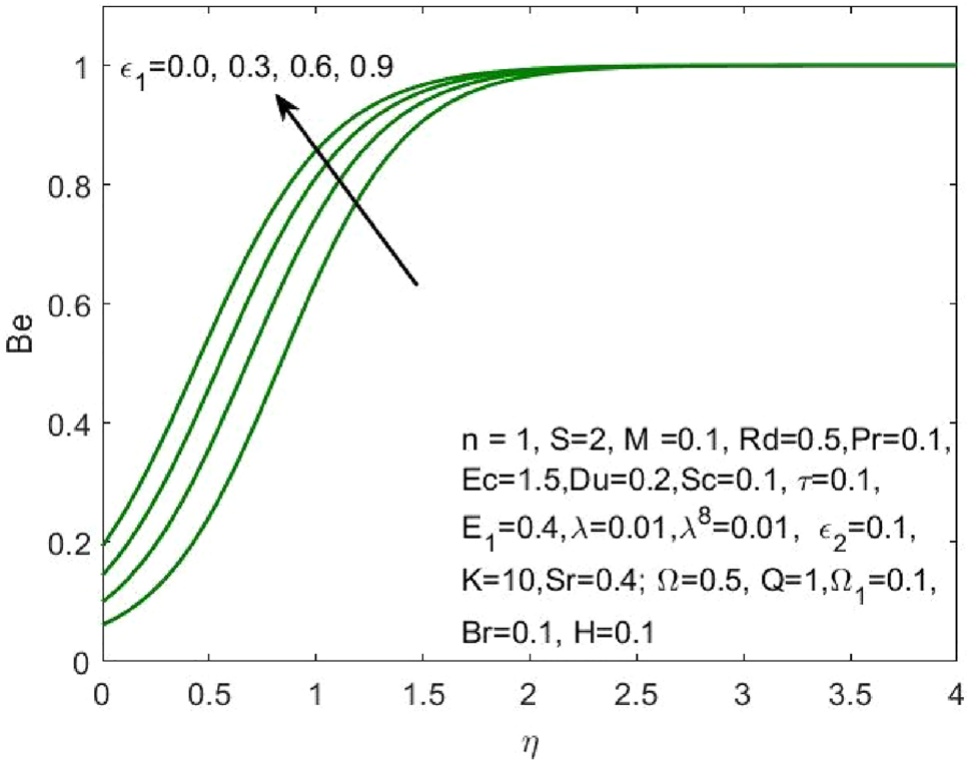
Influence of ${\varepsilon }_{1}$ on the $Be$.

**Figure 21:**
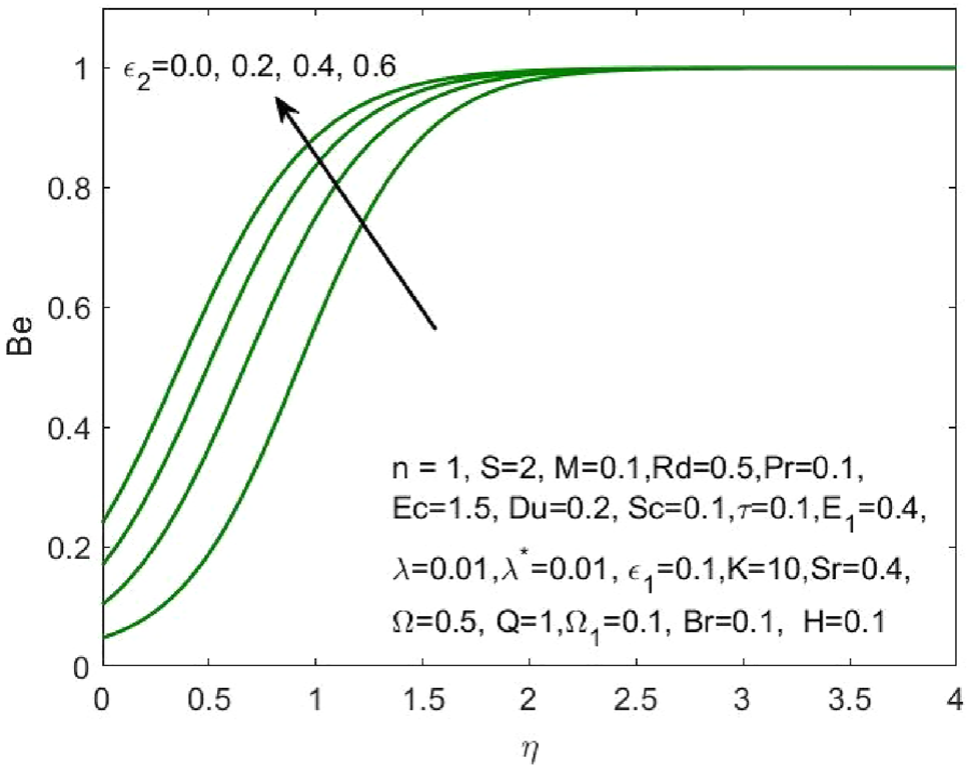
Influence of ${\varepsilon }_{2}$ on the $Be$.

The mathematical model framed using the PDEs as specified in (1)–(6) were transformed to ODEs (12)–(15) using the transformation given in (7). The subsequent system of transformed ODEs was solved by implementing the bvp4c package as described above with an accuracy of ${10}^{-6}$ and the solutions are validated by comparing with the existing literatures. The analysis is performed to understand the significant role of different features of fluid flow parameters on the mass and heat transfer profiles of the nanofluid. Table 1 and the graphical results (Figs. 2, 3, 4, 5, 6, 7, 8, 9, 10, 11, 12, 13, 14, 15, 16, 17, 18, 19, 20, 21) are used to report the model's outcomes. The graphs were obtained by varying one or two of the fluid parameters and keeping the rest as constants. The constant values of the parameters for this study were chosen to be: $n=1, S=2, M=0.1, R=0.1, Pr=6.2, Ec=0.1, Du=0.2, Sc=2, \tau =0.1, {E}_{1}=0.4, \lambda =0.1, {\lambda }^{*}=0.1, {\varepsilon }_{1}=0.1, {\varepsilon }_{2}=0.01, K=100, Sr=0.4, Q=1$.

**Table 1:** Numerical outcomes of $-\theta^ {\prime}(0)$ against diverse values of $Pr$ providing the validity of the current work.

$Pr$	Saba et al.^56^	Ishak et al.^57^	Mishra et al.^58^	Present study
	$$-\theta^ {\prime}(0)$$			
$0.72$	$0.80884$	$0.8086$	$0.8088$	$0.808688$
$1$	$1.00001$	$1.0000$	$1.0000$	$1.000018$
$3$	$1.92368$	$1.9237$	$1.9236$	$1.923599$
$7$	$3.07226$	$3.0723$	$3.0723$	$3.072316$
$10$	$3.72068$	$3.7207$	$3.7206$	$3.720589$
$100$	$12.29407$	$12.2941$	$12.2941$	$12.294108$

The effect of the both the slip parameters on the flow rate of the nanofluid along a stretching surface is depicted to be decreasing in the Figs. 2 and 3 respectively. The increase in these parameters creates a back flow at the boundary region which opposes the fluid. Moreover, ${\varepsilon }_{1}$ has opposite effect across a shrinking surface whereas ${\varepsilon }_{2}$ has same decreasing effect. Thus, the decrease is observed for higher values of slip parameters ${\varepsilon }_{2}$ across a stretching and shrinking surface whereas ${\varepsilon }_{1}$ possess opposite effect across a stretching and shrinking surface. Figure 4 illustrates the direct relationship between the curvature parameter K and the sheet radius. As the sheet radius lowers, less area is available for particles to adhere to, which in turn causes the stretching and shrinking rate to reduce and the fluid velocity to decrease. The higher values of mixed convection parameter signify a greater temperature gradient indicating a lighter density of fluid. Thus, it enables the fluid to flow at a faster velocity across a stretching surface as shown in Fig. 5. Although opposite effects were shown across a shrinking surface. The higher buoyancy ratio parameter reduces the temperature gradient and the concentration gradient increases. This makes the fluid flow difficult and hence a diminishing effect is observed in Fig. 6 for the increase in ${\lambda }^{*}$.

As the higher $Du$, there is an increase in temperature and thermal diffusion, as seen by the accelerating behavior of $\theta \left(\eta \right)$ in Fig. 7. The heat generated due to the internal friction occurring because of the flow boost with the rise in the Eckert number. The boost in the radiation signifies that the heat dissipated through the surface is more which is conducted by the nanofluid hence the amount of heat that a nanofluid conducts will be more as shown in Fig. 8. The growth in the suction parameter diminishes the fluid temperature as shown in Fig. 9. The upsurge in the Soret number signifies a greater temperature difference which shows that the temperature at the surface is evidently high than the surrounding. The temperature of the nanofluid upsurges overall as a result of the nanofluid absorbing this high heat, as depicted in Fig. 10.

As $Sr$ increases, the concentration profile $\phi \left(\eta \right)$ also increases, as seen in Fig. 11. The "effect of ratio of temperature difference to concentration difference" is what Soret number is defined as. This makes it clear that a larger concentration profile $\phi \left(\eta \right)$ is produced by diffusive species with higher Soret values. The increasing Schmidt number indicates that the concentration diffusion is more as compared to the previous stage, and this results in the decrease in the nanoparticle concentration profile as shown in Fig. 12. Enhancing the values of non-dimensional activation energy ${E}_{1}$ describes the cumulative response in the $\phi \left(\eta \right)$ as shown in Fig. 13. The mathematical relationship in Eq. (16) clarifies that the low temperature and high activation energy decreases the rate of chemical reaction, which trigger the chemical reaction process to slow down. As a result, the concentration $\phi \left(\eta \right)$ of the nanofluid enhances.

The ratio of the viscous dissipation to the external heating is termed Brinkman number and the increase in this value enhances the viscous dissipation and slows down the conduction of heat produced. Thus, decreasing the entropy generation as shown in Fig. 14. Similarly, the rise in the velocity slip parameters ${\varepsilon }_{1}$ and ${\varepsilon }_{2}$ enhances the local entropy generation as shown in Figs. 15 and 16 respectively.

**Figure 22:**
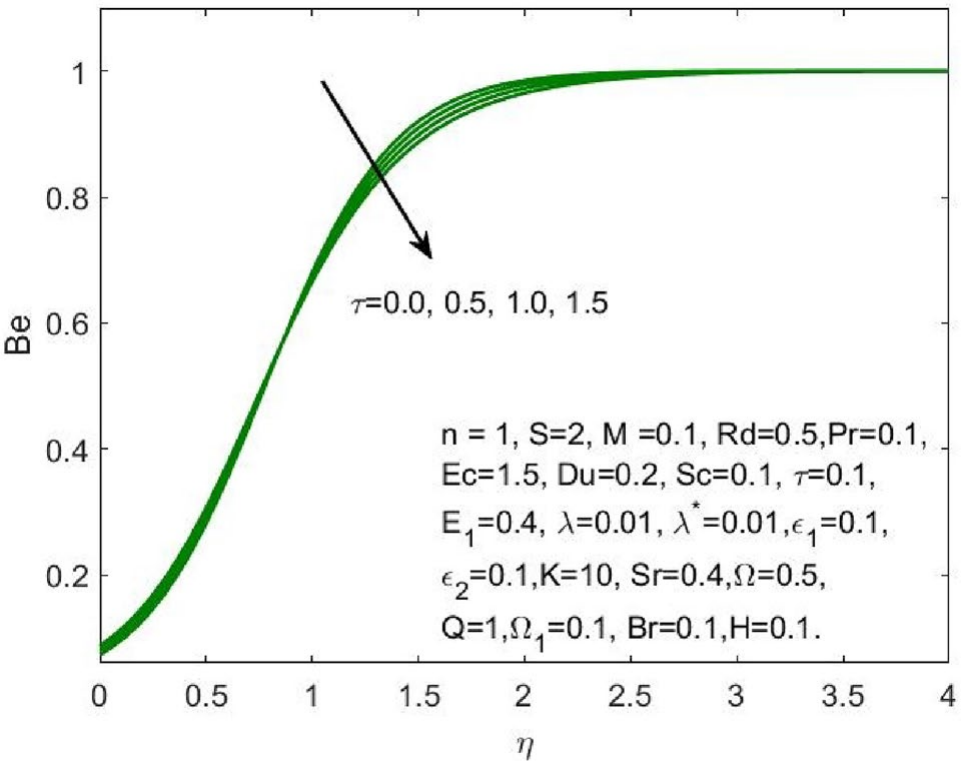
Influence of $\tau$ on the $Be$.

Figure 17 indicated that the rise in the parameter concerning the curvature of the radius shall diminish the entropy profile because of the change in the boundary’s physical configuration in the radial direction. The increasing values of $\omega$ signifies a larger difference in the concentration of nanoparticle which reduces the entropy profile as exposed in Fig. 18. With the rise in Brinkman parameter, the pressure drop also diminishes and as a consequence, a reduction in the Bejan number is recorded as shown in Fig. 19. Whereas the velocity slip parameters ${\varepsilon }_{1}$ and ${\varepsilon }_{2}$ increase the Bejan number as shown in Figs. 20 and 21 respectively. Meanwhile, the cumulative values of the chemical reaction parameter reduces the nanoparticle concentration which further reduces the pressure drop and as a consequence, the Bejan number declines effectively as seen in Fig. 22.

## Conclusion

The RKF-45 method is used to numerically analyze the Entropy generation for the flow of nanofluid across a curved stretching/shrinking surface. The mathematical model was framed by assuming the flow to be steady and laminar subjected to Magnetic field and mixed convection. Furhtermore the enerfy equation was designed using the cross diffusion model and the mass transfer equation is equipped with the Arrhenius activation energy. With these factors, the system of PDEs that governed the flow was transformed into a system of ODEs and the subsequent system of equations were solved by implementing RKF-45 method. The conclusions of the study indicate that:

- With increasing velocity slip parameters, there was a drop in the flow velocity, the Bejan number, and the local entropy generation.
- The higher values of the mixed convection parameter respectively increased and decreased the speed of the flow across a stretching and shrinking surface whereas the higher values of buoyancy ration parameter decreased the flow speed.
- A direct relationship is present between the fluid velocity and the curvature parameter.
- The Soret effects contributed in enhancing the total heat conduction by the nanofluid while the greater values of Eckert number showed a decreament in the thermal conduction.
- The higher Schmidt numbers decreased the mass transfer profile whereas the higher Soret numbers enhanced the concentration in the nanofluid.
- The increase in the Brinkman number showed increasing effects on the local entropy generation whereas it had a diminishing impact on the Bejan number.
- A reduction in velocity is detected for the rising values of slip parameter across a stretching and shrinking surface.
- The mathematical model presented in this article is completely based on the Nvier stokes equation which often assumes ideal conditions and might not reflect the real world situations. Therefore, this model can be adapted to understand the flow complexity and make necessary modifications as per the requirements.
- Enhancing the values of ${E}_{1}$ describes the cumulative response in the $\phi \left(\eta \right)$.

## Data Availability

Upon reasonable request, the datasets utilized and/or analyzed in the current work will be made available by the corresponding author.

## List of symbols

$${Re}_{s}$$: Reynolds number
$$\mu$$: Dynamic viscosity [$${\text{kg}}\;{\text{m}}^{ - 1} {\text{s}}^{ - 1}$$]
$$\varphi$$: Nanoparticle’s concentration
$$\nu$$: Kinematic viscosity [$${\mathrm{m}}^{2}/\mathrm{s}$$]
$s$: Arc length coordinate with respect to the curved surface
$$(\rho {C}_{p})$$: Heat capacity
${\lambda }^{*}$: Buoyancy ratio parameter
$$\alpha$$: Thermal diffusivity $$[{\mathrm{m}}^{2}{\mathrm{s}}^{-1}]$$
${C}_{{f}_{s}}$: Coefficient of skin friction
$${Sh}_{s}$$: Sherwood Number
$${Nu}_{s}$$: Nusselt Number
*p*: Dimensional pressure [$${\text{kg}}\;{\text{m}}^{ - 1} {\text{s}}^{ - 2}$$]
$T$: Temperature of the fluid [$\mathrm{K}$]
$$\kappa$$: Thermal conductivity $$[{\mathrm{Wm}}^{-1}{\mathrm{K}}^{-1}]$$
$a$: Constant related to stretching and shrinking of the sheet
$$\rho$$: Density [$${\mathrm{kgm}}^{-3}$$]
${\tau }_{rs}$: Wall shear stress
$${\sigma }_{f}, {\sigma }_{s}$$: Electrical conductivity [$${\mathrm{Sm}}^{-1}$$]
$\lambda$: Mixed convection parameter
$$S$$: Suction parameter
$K$: Dimensionless curvature parameter
$u, v$: Velocity components in $s, r$ directions respectively [${\mathrm{ms}}^{-1}$]
$R$: Curvature of the curves belt
$C$: Concentration of the fluid [$\mathrm{mol} {\mathrm{m}}^{-3}$]
${C}_{w}, {C}_{\infty }$: Concentration near and far away from the surface respectively
${T}_{w}, {T}_{\infty }$: Surface temperature and temperature far away from the surface respectively
$r$: Normal to the tangent at any point of the curved surface
$M$: Magnetic field parameter
${B}_{0}$: Magnetic field strength [$\mathrm{T}$]
$${D}_{m}$$: Molecular diffusivity [$${\mathrm{m}}^{2}{\mathrm{s}}^{-1}$$]
$$Pr$$: Prandtl number
${j}_{w}$: Wall heat flux
$${k}_{1}$$: Boltzmann constant [$$8.314\; \mathrm{J}/\mathrm{K} \mathrm{mol}$$]
$${\nu }_{w}$$: Suction velocity
$n$: Fitted rate constant [W ${\mathrm{m}}^{-2}{\mathrm{K}}^{-1}$]
${k}^{*}$: Mean absorption coefficient [${\mathrm{m}}^{-1}$]
$$P$$: Dimensionless pressure
$$Ec$$: Eckert number
$${c}_{s}$$: Concentration susceptibility [$${\mathrm{kgm}}^{-3}$$]
${q}_{w}$: Wall heat flux
${k}_{r}$: Chemical reaction rate [$\mathrm{mol} \;{\mathrm{L}}^{-1}{\mathrm{s}}^{-2}$]
${\sigma }^{*}$: Stefan–Boltzmann constant [${\mathrm{Wm}}^{-2}{\mathrm{K}}^{-4}$]
$${E}_{a}$$: Activation energy [$$\mathrm{J}$$]
${k}_{T}$: Thermal diffusion ratio
$$f$$: Stream function
$$\eta$$: Similarity variable
$${f}^{\prime}$$: Dimensionless velocity
$\theta$: Dimensionless temperature of fluid
$\phi$: Dimensionless concentration of fluid
$$L$$: Slip length
$$Br$$: Brinkman number
$$H$$: Diffusion parameter
$$Rd$$: Radiation parameter
$$Du$$: Dufour number
$$Sc$$: Schmidt number
${E}_{1}$: Dimensionless activation energy parameter
$\omega$: Temperature difference parameter
$\varepsilon$: Dimensionless slip length
$\tau$: Dimensionless chemical reaction rate parameter
$${\omega }_{1}$$: Concentration difference
${\varepsilon }_{1}$, ${\varepsilon }_{2}$: Velocity slip parameter
$$Sr$$: Soret number
${N}_{G}$: Local entropy generation
$\gamma$: Stretching/shrinking parameter
